# Transcriptome Analysis Reveals Norathyriol Prolongs the Lifespan via Regulating Metabolism in *C. elegans*

**DOI:** 10.3390/metabo14120716

**Published:** 2024-12-19

**Authors:** Hong-Jia Zhang, Hai-Quan Lan, Meng-Ying Wang, Cai-Feng Wang, Lu-Gang Wei, Chen Xu

**Affiliations:** 1 State Key Laboratory of Pharmaceutical Biotechnology, School of Life Sciences, Nanjing University, Nanjing 210023, China; 522022300048@smail.nju.edu.cn (H.-J.Z.); 502022300090@smail.nju.edu.cn (H.-Q.L.); 522022300039@smail.nju.edu.cn (M.-Y.W.); 18878868761@aliyun.com (L.-G.W.); 2 State Key Laboratory of Materials-Oriented Chemical Engineering, College of Chemical Engineering, Nanjing Tech University, Nanjing 210009, China; caifengwang@njtech.edu.cn

**Keywords:** norathyriol, aging, *C. elegans*, lipid metabolism, transcriptome analysis

## Abstract

Background: Aging and age-related diseases are closely linked to an imbalance in energy supply and demand, a condition that can potentially be mitigated through various interventions, including the use of naturally occurring molecules. Norathyriol (NL), a tetrahydroxyxanthone compound, is prevalent in mango fruit and medicinal plants. While studies have indicated that NL may influence metabolism, its effects on aging have not been extensively explored. Methods: We conducted lifespan analysis and measured lipofuscin accumulation in *C. elegans* model to evaluate the effects of NL on aging. Additionally, we identified differentially expressed genes (DEGs) through comprehensive RNA-sequencing (RNA-seq) analysis and performed gene ontology (GO) and kyoto encyclopedia of genes and genomes (KEGGs) pathway analyses to elucidate the molecular mechanisms underlying NL’s effects. Results: Our study demonstrated that NL at 50 μM extends the lifespan by 15.9% and reduces lipofuscin accumulation in *C. elegans* without impacting their feeding capabilities. A total of 928 DEGs were identified in NL-treated worms. The analysis of DEGs indicated that NL’s longevity-promoting effects might be due to its regulation of gene expression in lipid metabolism and immune response pathways. Furthermore, the insulin/insulin-like growth factor (IGF)-1 and target of rapamycin (TOR) signaling pathways were implicated in the lifespan-extending effect of NL. Conclusions: These findings broaden the bioactivity profile of polyphenols and highlight the need for further investigation into the therapeutic potential of NL in combating age-related diseases.

## 1. Introduction

Aging is mainly manifested as the gradual decline of the biological function of the organism and eventually leads to the death of the organism. In humans, aging also contributes to a variety of age-related diseases, including cancer, diabetes, cardiovascular disease, and neurodegenerative diseases [1]. A growing body of evidence suggests that aging and age-related diseases are closely linked to an imbalance in energy supply and demand [2,3,4]. Mitochondria regulate the cellular energy supply by activating the metabolism of carbohydrates and fats, thereby playing an essential role in aging and age-related diseases. The imbalance between energy supply and demand can potentially be reduced through various strategies, such as engaging in physical exercise [5] and limiting caloric intake [6], as well as by using naturally occurring compounds that affect longevity pathways, including metformin [7], resveratrol [8], and rapamycin [9].

Norathyriol (NL) is the primary metabolite of mangiferin, possessing a chemical structure of tetrahydroxyxanthone. It is commonly found in the fruit of *Garcinia mangostana* and in medicinal plants from the *Guttiferae* and *Gentianaceae* families, including *Hypericum perforatum*, also known as St. John’s Wort. Studies have shown that NL has beneficial effects against a variety of disease conditions, such as cancer, diabetes, and cardiovascular disease [10,11,12]. Of particular note, NL was reported to inhibit a-glucosidase in vitro and in vivo [13] and increase sirtuin-1 and AMPK phosphorylation in mice [14]. Moreover, NL was reported to reverse obesity- and high-fat-diet-induced insulin resistance in mice through inhibition of PTP1B [10]. While studies have indicated that NL may influence metabolism, its effects on aging have not been extensively explored.

In this study, we aimed to investigate the impact of NL on lifespan and the accumulation of lipofuscin in *Caenorhabditis elegans*, a widely used model organism for aging research. To elucidate the global transcriptomic shifts in response to NL and to uncover the underlying anti-aging mechanisms, we employed RNA sequencing (RNA-seq). This robust technique allows for a comprehensive examination of transcriptome-wide differential gene expression and the intricate architecture of the transcriptome [15,16]. It encompasses the identification of novel transcripts, the detection of allele-specific expression, and the characterization of splice junctions [17,18]. We revealed that NL could extend lifespan and reduce lipofuscin accumulation in *C. elegans*. In NL-treated worms, a total of 928 differentially expressed genes (DEGs) were identified, suggesting that NL’s lifespan-extending effects may be mediated through its influence on lipid metabolism, immune response, and key signaling pathways associated with aging. Overall, these findings provide clues for future in-depth research on the mechanisms by which NL extends lifespan and may offer a scientific basis for the development of anti-aging interventions.

## 2. Materials and Methods

### 2.1. C. elegans Strains and Culture

The *C. elegans* strains employed in this research were sourced from the Caenorhabditis Genetics Center, supported by the NIH Office of Research Infrastructure Programs. The strain included in this study is N2 (wild type). The worms were routinely cultivated and maintained on standard nematode growth media (NGM) seeded with *Escherichia coli* OP50 at 20 °C.

### 2.2. Chemicals

Norathyriol (NL), with a purity level exceeding 95% as determined by HPLC (Agilent, Santa Clara, CA, USA), was synthesized through the deglycosylation of mangiferin and confirmed by mass spectrometry (MS) (Agilent, Santa Clara, CA, USA) and nuclear magnetic resonance (NMR) (Bruker, Billerica, MA, USA) [19].

### 2.3. Lifespan Analysis

The lifespan assays were conducted as described previously with slight modifications [20]. Briefly, synchronized worms were grown on NGM plates seeded with OP50 at 20 °C until they reached the young-adult stage. Subsequently, they were transferred to fresh NGM plates seeded with OP50 and treated with 5 μM 5-fluoro-2′-deoxyuridine (FUdR, Sigma, St. Louis, MO, USA), either with or without the addition of 50 μM NL. To ensure an adequate food supply and minimize the risk of mold contamination, the worms were moved to fresh assay plates every 4 days.

Around 120 worms per condition were monitored for mortality every 2 or 3 days until all had died. All lifespan experiments were carried out at 20 °C. Censoring was applied in cases where worms became dehydrated on the plate’s edge, escaped, burst, or experienced internal hatching. Statistical analysis was performed using Oasis 2 (Online Application of Survival analysis, http://sbi.postech.ac.kr/oasis, accessed on 22 April 2022), and *p* values were calculated using the log-rank (Mantel-Cox) test method.

### 2.4. Lipofuscin Accumulation

Lipofuscin assays were executed with minor adjustments as previously described [21]. Synchronized young-adult worms were placed on NGM plates treated with 5 μM FUdR, with or without 50 μM NL, and allowed to develop until the eighth day of adulthood. The worms were then picked and mounted on a 2% agarose pad coated with 1 mM levamisole (Sigma, St. Louis, MO, USA). The autofluorescence of lipofuscin in the worms’ gut was captured using a fluorescence microscope (CX40, Shun Yu, Ningbo, China). The fluorescence intensity was quantified by ImageJ software (v1.54d, Rasband, W.S., ImageJ, U.S. National Institutes of Health, Bethesda, MD, USA, https://imagej.net/ij/, accessed on 22 April 2022).

### 2.5. Feeding Rate Assay

The feeding rate assays were conducted with some adjustments as previously described [22]. In brief, synchronized young-adult nematodes were transferred to NGM plates treated with 5 μM FUdR and containing 50 μM NL and allowed to grow until they reached day 1 and 9 of adulthood. Pharyngeal pumping (feeding) rates were then counted for 10 s by observing the worm’s pharynx under a dissecting microscope (SMZ-168, Motic, Xiamen, China) and converted to the number of pumps per minute.

### 2.6. Transcriptome Sequencing and Analysis of RNA Seq Data

Synchronized young-adult wild-type worms were placed on NGM plates treated with 5 μM FUdR, either with or without 50 μM NL. After a 24-h incubation at 20 °C, the worms were collected by washing with M9 buffer three times and then flash-frozen in liquid nitrogen. mRNA-seq experiments were performed using three independent biological replicates for each condition.

Total RNA from *C. elegans* was extracted using TRIzol^®®^ Reagent (Invitrogen, CA, USA). RNA quality was assessed using a Bioanalyser (Agilent2100, CA, USA). Sequencing libraries were prepared using the TruSeq™ RNA Sample Prep Kit (Illumina, CA, USA), and paired-end sequencing was carried out on an Illumina HiSeq 4000 platform (Meiji Biotechnology Co., Ltd., Shanghai, China).

The raw paired-end reads were processed and quality-checked using SeqPrep and Sickle (v1.2). The clean reads were then aligned individually to the *C. elegans* genome using Hisat2 (v2.1.0) [23], and the mapped reads from each sample were assembled using StringTie (v1.3.3b) [24]. Genes with aligned pairs were quantified using RSEM (v1.2.31) [25].

The raw counts were utilized for the identification of differentially expressed genes (DEGs). DEGs were identified as those with a fold change >2 and *p* value < 0.05 using DESeq2 (v1.22.2) [26].

Volcano plots were generated to compare gene expression in NL-treated worms with that in wild-type worms. Genes that were significantly induced or repressed by NL treatment were highlighted. Volcano plots and heatmaps were created using R and TBtools (https://cj-chen.github.io/tbtools/, accessed on 19 November 2024), respectively.

Gene ontology analysis and Kyoto encyclopedia of genes and genomes (KEGG) pathway enrichment analyses were performed using KOBAS (http://bioinfo.org/kobas, accessed on 16 November 2024) [27]. DEGs were significantly enriched in GO terms or KEGG pathways when their *p* value < 0.05.

All data plotting and statistical testing were carried out using R (v.4.2.3, http://www.r-project.org, accessed on 16 November 2024) unless otherwise indicated.

### 2.7. Quantitative RT-PCR Analysis

Quantitative RT-PCR was performed with minor adjustments as previously described [28]. Briefly, synchronized young-adult worms were placed on NGM plates treated with 5 μM FUdR, either with or without 50 μM NL, and incubated for 24 h. The worms were then washed and collected, and approximately 500–1000 individuals were used for qRT-PCR analysis. Total RNA was extracted using TRIzol reagent (Invitrogen, Carlsbad, CA, USA). Quantitative PCR was conducted using SYBR Green Master Mix (Applied Biosystems, Foster City, CA, USA) on a StepOnePlus Real-Time PCR System (Applied Biosystems, Foster City, CA, USA). The data were analyzed using the comparative 2-∆∆Ct method with ama-1 as the endogenous control. The primers used for qRT-PCR are detailed in Supplementary Table S2.

### 2.8. Oil Red O Staining

Oil Red O staining was executed with minor adjustments as previously described [29]. In brief, synchronized young-adult nematodes were transferred to NGM plates treated with 5 μM FUdR and containing 50 μM NL and incubated for 7 days at 20 °C. Approximately 100 worms were then harvested, washed, and fixed with a 40% isopropanol solution for 3 min. The fixed worms were incubated in a 60% Oil-Red-O solution at room temperature for 2 h. The stained worms were then washed three times with a PBST solution (PBS: 137 mM NaCl, 2.7 mM KCl, 10 mM Na2HPO4, 2 mM KH2PO4; PBST: PBS with 0.01% Triton X-100). Images were captured using a fluorescence microscope (CX40, Shun Yu, China) and quantified using ImageJ software (Rasband, W.S., ImageJ, U.S. National Institutes of Health, Bethesda, MD, USA, https://imagej.net/ij/, 1997−2009, accessed on 22 April 2022).

### 2.9. Statistical Analysis

Statistical analysis of the data, except lifespan data, was conducted using a two-tailed Student’s *t*-test unless otherwise specified, *p* value < 0.05 was considered to indicate statistical significance.

## 3. Results

### 3.1. NL Extends Lifespan and Reduce Lipofuscin Accumulation in C. elegans

**Figure 1:**
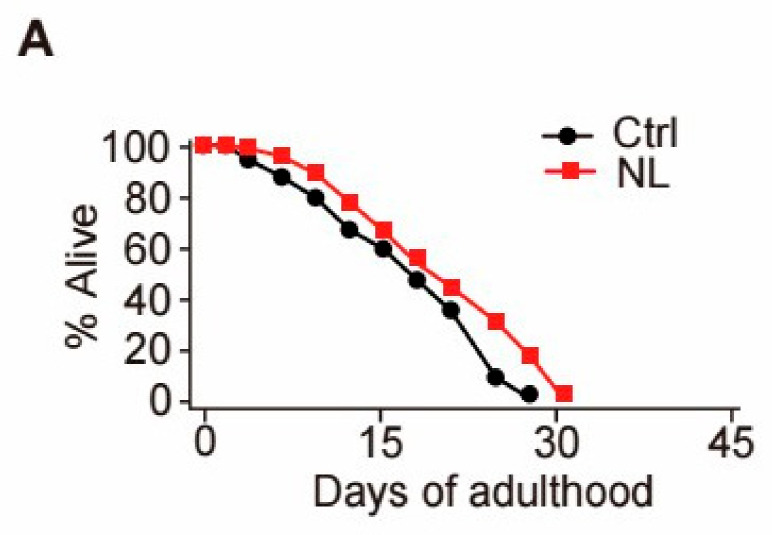

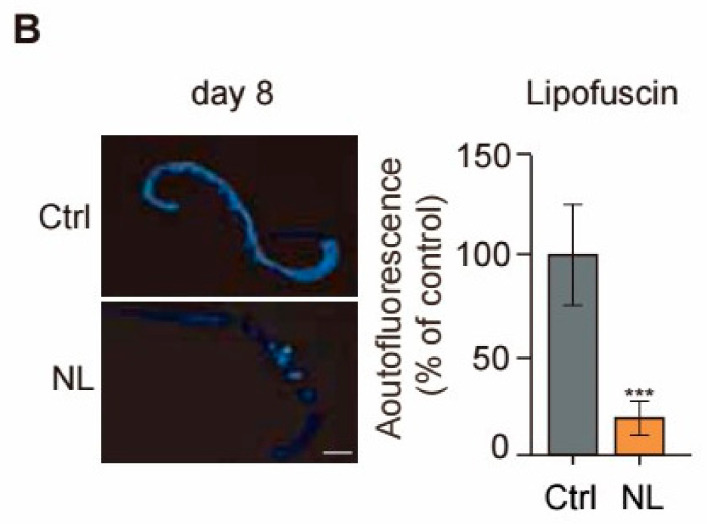
NL extends lifespan and reduces lipofuscin accumulation in *C. elegans*. (**A**) Effects of NL on lifespan in *C. elegans*. Synchronized worms at young-adult stage were treated with 50 μM NL (** *p* < 0.01, log-rank (Mantel-Cox) test). (**B**) Effects of NL on lipofuscin accumulation in *C. elegans*. The scale bar shows 100 μm. ImageJ was used to quantify the fluorescence in the left panel (*n* = 25 for three independent experiments). Data in bar graphs are expressed as mean ± SEM. (*** *p* < 0.001, two-tailed Student’s *t*-test).

To investigate the effect of NL on the lifespan of *C. elegans*, we conducted lifespan experiments and found that the average lifespan of nematodes treated with NL at 50 μM was significantly increased by 15.9% when compared to the control group (Figure 1A). As a result, unless otherwise specified, the same concentration was used in subsequent experiments. *C. elegans* intestinal lipofuscin accumulates with age and can be used to assess health or the rate of aging. We found that NL significantly reduced lipofuscin levels in aged worms (Figure 1B). These results showed that NL treatment delayed aging in *C. elegans*.

### 3.2. NL Has No Impact on the Feeding Capabilities of C. elegans

**Figure 2:**
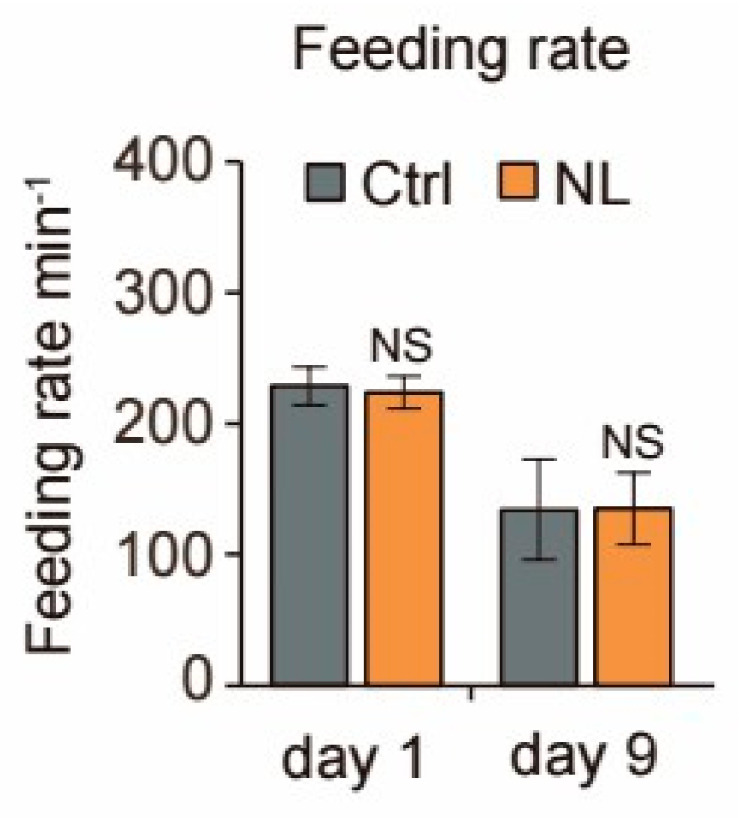
NL has no impact on the feeding capabilities of *C. elegans*. Effects of NL on feeding capabilities in *C. elegans*. Synchronized worms at young-adult stage were treated with 50 μM NL. Data in bar graphs are expressed as mean ± SEM. (two-tailed Student’s *t*-test).

Since NL treatment for the whole-life period of worms could extend worms’ lifespans, the impact from the food intake amount should be in consideration. To eliminate the potential influence of dietary restriction on the lifespan of *C. elegans*, we examined their feeding patterns. The results showed that there were no significant differences in food intake between the control and NL-treated groups in either 2 days or 9 days (Figure 2), indicating that NL-induced lifespan extension was not related to the diet-restrict pathway.

### 3.3. Differentially Expressed Genes (DEGs) Affected by NL in C. elegans

**Figure 3:**
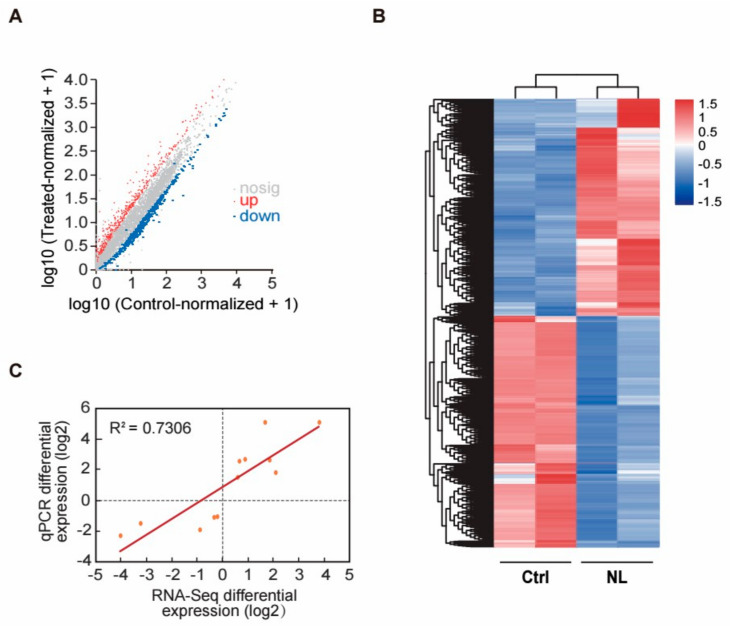
Volcano plot, heatmap, and qPCR analysis of genes altered by NL treatment in *C. elegans*. (**A**) Volcano plots showing the results of RNA-Seq; red dots represented 446 upregulated DEGs, and blue dots represented 482 down-regulated DEGs. (**B**) Heatmap of the genes significantly altered after NL treatment. Color correlates with the value of z-score. Z-score = (X − mean)/SD. (**C**) Confirmation of RNA-Seq results by qPCR with the upregulated or downregulated 13 genes exhibiting the largest fold change (R^2^ = 0.7306).

To delve deeper into the underlying mechanisms of how NL treatment extends lifespan, we conducted a comprehensive RNA-seq analysis. The volcano plots revealed that the administration of NL resulted in alterations in the expression levels of 928 genes in *C. elegans* compared to the controls, with 446 genes being upregulated and 482 genes being downregulated (Figure 3A). DEGs (*p* < 0.05) were presented in a heatmap (Figure 3B) and shown in Supplementary Table S1, indicating an expression profiling with a large number of genes altered after NL treatment.

Then quantitative polymerase chain reaction (qPCR) was performed to determine the mRNA levels of 12 genes selected from the up- and down- regulated DEGs to verify the RNA-Seq data. The results verified RNA-Seq output with an R^2^ of 0.7306, indicating the reliability of our RNA-seq data (Figure 3C).

### 3.4. The Lifespan-Extending Effect of NL Are Related to Insulin/IGF-1 and TOR Signaling Pathways

**Figure 4:**
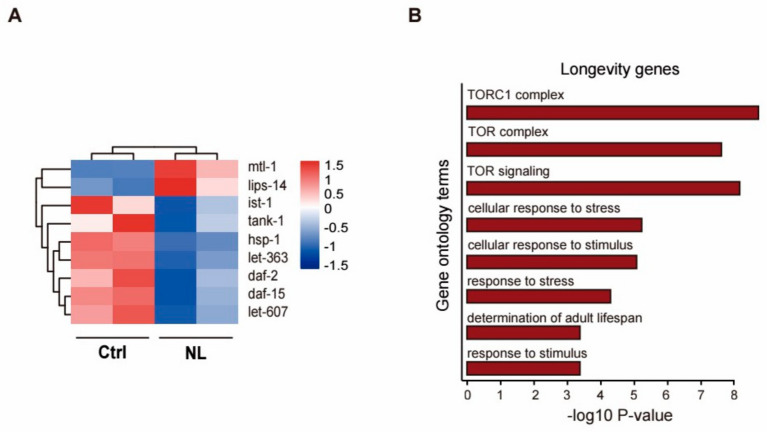
Heatmap and enrichments of GO terms associated with longevity genes altered by NL treatment in *C. elegans.* (**A**) Heatmap of significantly altered longevity-related genes after NL treatment. Color correlates with the value of z-score. Z-score = (X − mean)/SD. (**B**) Significant GO terms of longevity-related DEGs found by enrichment analyses in 50 μM NL-treated worms.

In order to identify the molecular pathways underlying the life-span extending effects of NL, we analyzed the DEGs by using the Majorbio Cloud Platform. We observed 9 genes among the DEGs were closely associated with longevity, as shown in Table 1 and presented in a heatmap (Figure 4A). Among them, the *ist-1* and *daf-2* genes are components of the insulin/IGF-1 signaling pathway. NL downregulated the expression of these genes, suggesting that NL may extend lifespan by attenuating the insulin/IGF-1 signaling pathway.

In particular, *let-363*, encoding components of the TORC2 complex in *C. elegans*, was also downregulated, suggesting that the TOR signaling pathway was suppressed. Additionally, GO enrichment analysis of the DEGs relevant to longevity has shown significant enrichment of terms related to the TOR signaling pathway, such as ‘TORC1 complex’, ‘TOR complex’, and ‘TOR signaling’. This finding, along with the downregulated *let-363* expression, implies that NL could prolong lifespan potentially by dampening the TOR signaling pathway (Figure 4B).

Collectively, these results suggest that NL exerts its lifespan-extending effects by modulating both the insulin/IGF-1 and TOR signaling pathways.

### 3.5. Enrichments of GO Terms and KEGG Pathways in the Transcriptomes of NL-Treated C. elegans

We next examined the biological function altered by NL treatment in *C. elegans* and studied the gene expression profile from RNA-seq by using KEGG and GO term enrichment analyses.

**Figure 5:**
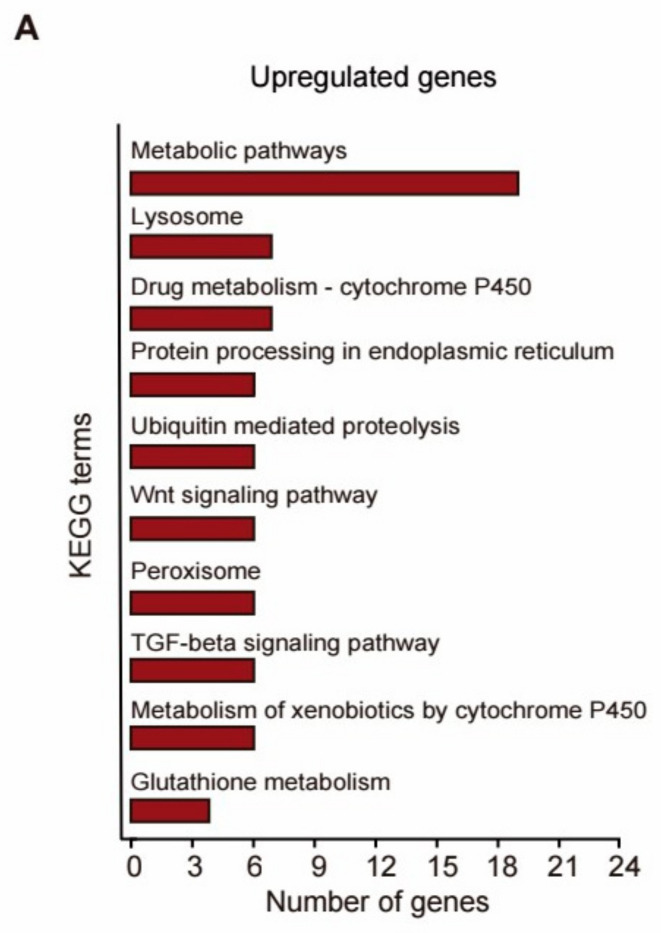

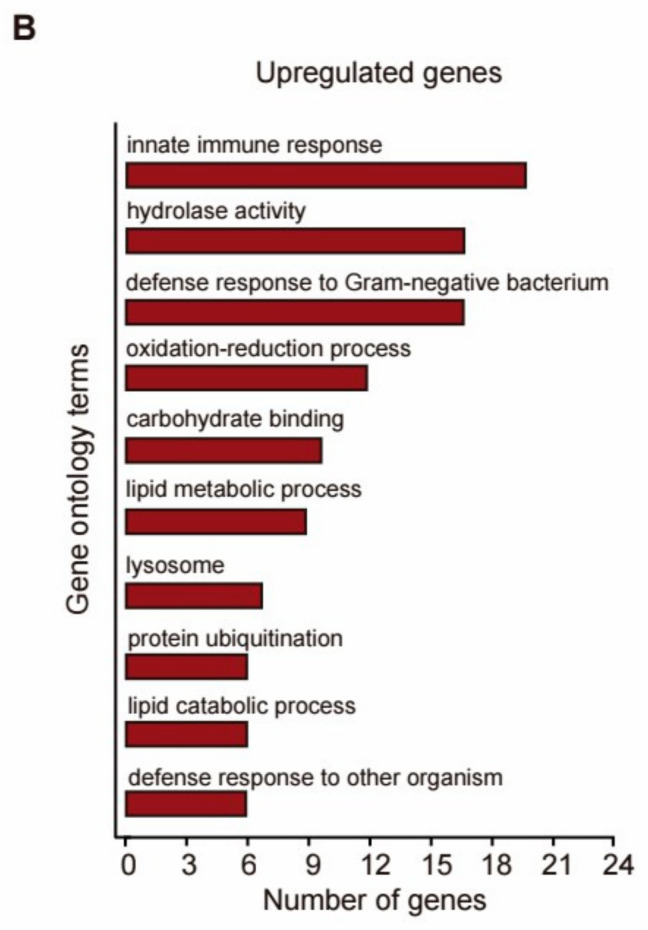
Enrichments of GO terms and KEGG pathways in the transcriptomes of NL-treated worms. (**A**) KEGG analysis of genes significantly upregulated in NL-treated worms. (**B**) Significant GO terms of upregulated DEGs found by enrichment analyses in 50 μM NL-treated worms.

KEGG analysis shown in Figure 5A revealed significant alterations in a total of 22 KEGG pathways in response to NL treatment (*p* < 0.05) (Supplementary Table S3). Notably, metabolic pathways such as Metabolic pathways, Lysosome, Peroxisome, Metabolism of xenobiotics by cytochrome P450, and Glutathione metabolism were among the most prominently enriched pathways. Cytochrome P450 (CYPs), a family of heme-thiolate proteins, engage in numerous vital biological processes, including xenobiotic metabolism, regulation of fatty acids, dauer formation, and stress response in *C. elegans* [30]. The TGF-β signaling pathway holds a significant correlation with both immune responses and the aging process [31]. The Wnt signaling pathway exhibits a profound association with the senescence process in *C. elegans* [32]. NL appears to regulate these signaling pathways, ultimately contributing to the promotion of longevity in NL-treated *C. elegans*.

GO term enrichment analysis identified 67 significant terms that were altered by NL treatment (Supplementary Table S4). The most highly represented GO terms affected by NL treatment included “Innate immune response”, “Defense response to Gram-negative bacterium”, “Defense response to other organism” and “Defense response to bacterium.” These biological function alterations are known to influence immune responses (Figure 5B). Furthermore, a significant enrichment was observed in genes associated with “Hydrolase activity”, “Carbohydrate binding”, “Lysosome” and “Protein ubiquitination”, all of which have been shown to impact longevity. It is also noteworthy that genes enriched in “Oxidation-reduction process”, “Lipid metabolic process” and “Lipid catabolic process” were predominantly linked to lipid and fatty acid metabolism.

Taken together, both KEGG and GO enrichment analyses highlighted the role of metabolism in NL-mediated longevity. Also, the longevity promotion mechanisms of NL may be related to its modulation of gene expression in immune response.

### 3.6. Effects of NL on Lipid Metabolism in C. elegans

The above enrichment analyses suggest that NL treatment might influence metabolism by altering the transcriptional profile of the worms. Among them, lipid metabolism plays a crucial role in the aging process, especially in the longevity mediated with diet, genetics, or pharmacological interventions in various model organisms [33,34,35]. We therefore applied the Majorbio Cloud Platform to analyze the DEGs and examined fat accumulation in NL-treated worms by using Oil Red O staining.

**Figure 6:**
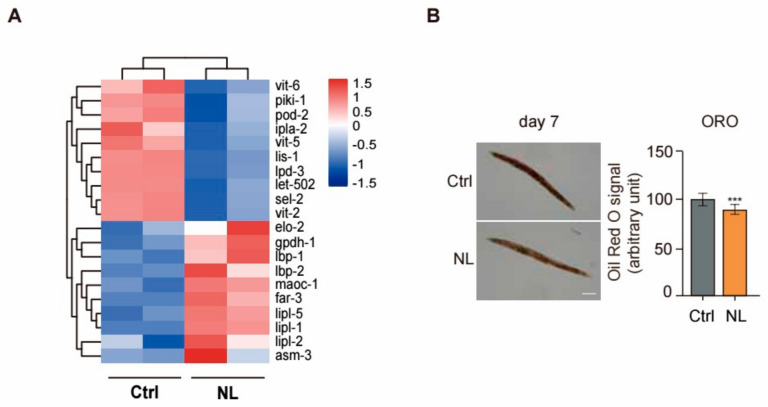
Effects of NL on fat accumulation in *C. elegans.* (**A**) Heatmap of significantly altered lipid-related genes after NL treatment. Color correlates with the value of z-score. Z-score = (X − mean)/SD. (**B**) Effects of 50 μM NL on fat accumulation in *C. elegans*. The scale bar shows 100 μm. Quantification of the Oil red O staining data is shown in the right panel (*n* = 25 for three independent experiments). Data in bar graphs are expressed as mean ± SEM. (*** *p* < 0.001, two-tailed Student’s *t*-test).

We found that 20 genes in RNA-seq DEGs data were associated with lipid metabolism, as presented in a heatmap (Figure 6A). The heatmap revealed that NL treatment significantly upregulated the expression of genes pivotal to lipid catabolism, including *lipl-1*, *lipl-2*, and *lipl-5*, all belonging to the lipase family. Meanwhile, NL treatment significantly suppressed the expression of genes indispensable for fatty acid synthesis, such as *ipla-2* and *pod-2*. Moreover, the NL treatment decreased the expression of genes related to vitamin metabolism, including *vit-2*, *vit-5*, and *vit-6*. Vitamins are engaged in lipid anabolic reactions as coenzymes or cofactors. Accordingly, the reduced expression of genes engaged in vitamin metabolism may perturb lipid synthesis metabolism, thereby improving lipid degradation (Figure 6A).

The similar result was observed in the Oil Red O staining experiment. A significant reduction in fat accumulation was observed in worms 7 days after NL exposure (Figure 6B), indicating that NL effectively reduces fat accumulation in *C. elegans*.

Thus, these findings demonstrated that NL treatment curbed lipid accumulation in *C. elegans* by realigning the metabolic balance in favor of lipid catabolism.

## 4. Discussion

In this report, we provide the first evidence showing that NL can delay aging in *C. elegans* model. Furthermore, utilizing RNA-sequencing analysis, we elucidated that NL’s anti-aging effects are attributed to its capacity to modulate key genes within lipid metabolism, immune response, as well as insulin/IGF-1 signaling and TOR pathways.

### 4.1. NL Prolongs the Lifespan

NL structurally belongs to xanthone, a subgroup of naturally occurring flavonoids. It has been reported to possess activities against a variety of disease conditions, such as cancer, diabetes, and cardiovascular disease. Among them, its activities in reversing diabetes and regulating lipid metabolism have attracted great attention. In the present study, we first found that NL has the ability to delay aging, manifested with the extended lifespan as well as the decreased lipofuscin accumulation in *C. elegans* after NL administration.

To the best of our knowledge, polyphenols, a class of plant secondary metabolites widely present in nature, are structurally divided into hydroxybenzoic acid, hydroxycinnamic acid, flavonoids, and lignins. As the largest subclass of polyphenols, flavonoids hold great potential in aging research due to their potent antioxidant and anti-inflammatory properties [36]. For example, flavonols such as quercetin, kaempferol, and apigenin; flavanones like hesperidin; and isoflavones including genistein and daidzein have all been reported to possess anti-aging properties [37]. However, as for the chemical class of xanthone, there is limited literature on aging research, with the exception of reports indicating that mangiferin, a well-known xanthones, has been shown to ameliorate age-related cardiac decline in D-galactose-induced aging rats [38]. In this regard, our findings may expand the bioactivity profile of polyphenols.

### 4.2. NL’s Lifespan-Extending Effect Is Associated with Insulin/IGF-1 and TOR Signaling Pathways

The insulin/IGF-1 signaling pathway is intricately linked to the aging process, exerting its influence on lifespan by modulating cellular growth, differentiation, metabolism, and the response to stress [39,40]. Evidence from various model organisms, including yeast, *C. elegans*, and fruit flies, has consistently demonstrated that the inhibition of critical components within the insulin/IGF-1 signaling pathway can lead to an extension of lifespan [41,42,43]. Moreover, this pathway is also implicated in the development of metabolic disorders such as diabetes and obesity, which are known to accelerate the aging process [44]. Our data indicated that NL modulates the expression of several genes associated with the insulin/IGF-1 pathway, notably downregulating the expression of *daf-2*, a key receptor in the insulin/IGF-1 signaling pathway. It has been established that diminishing DAF-2 activity can double the lifespan of *C. elegans* [41]. Thus, our data suggest that NL exerts its lifespan-extending effects by attenuating the insulin/IGF-1 signaling pathway.

Additionally, our data showed that NL significantly reduced transcriptional expression of *let-363*, encoding components of the TORC2 complex in *C. elegans*, indicating that the TOR pathway was suppressed. The TOR signaling pathway, participating in nutrient sensing, is also involved in aging [45]. In mice, the deletion of genes encoding the TORC1 subunit can extend lifespan [46]. Direct inhibition of the TOR signaling pathway by rapamycin has beneficial effects during aging. The present result showed that NL-mediated longevity was partly through suppression of TOR signal, raising a possibility of NL as a potential inhibitor of the TOR pathway.

### 4.3. NL Modulates Gene Expression in Lipid Metabolism and Immune Response

To further explore how NL promotes longevity, we performed RNA-seq on *C. elegans* treated with NL and analyzed the transcriptomic changes. Following NL treatment, transcriptional expression profiling indicated substantial changes in the expression levels of numerous genes. NL seems to modulate a broad spectrum of genes that are pivotal to signaling pathways and biological processes associated with aging and longevity.

The enrichment analyses of GO terms and KEGG pathways demonstrated that the most prominent functions of the genes altered by NL treatment were related to metabolism, immune response, and aging pathways. It is well known that metabolism, immune response, and aging are closely related [47,48].

A growing number of literatures indicate that aging is correlated with a decline in immune functions. Concurrently with these functional changes, the most dramatic gene expression changes that occur during aging are implicated in the immune response [49]. Additionally, a wealth of evidence confirms that aging and age-related diseases are closely linked to an imbalance in energy supply and demand [2,3,4]. Recent studies have underscored the pivotal role of mitochondria in cellular immunity. Notably, research has shown that mitochondrial chaperones, such as HSP-60, can enhance host eukaryotic resistance to pathogenic bacteria by up-regulating cytosolic p38 MAPK signaling, a pathway that is evolutionarily conserved across species [50].

In our study, it seems that NL elicited substantial transcriptional alterations within the metabolic and immune response pathways, which are pivotal for the modulation of lifespan, ultimately contributing to the observed extension of longevity in NL-treated *C. elegans*. These findings provide valuable clues for future in-depth research on the mechanisms by which NL extends lifespan.

Significantly, the DAF-16/FOXO transcription factor, a key component of the insulin/IGF-1 signaling pathway, has been identified as a crucial immune regulator in the defense against bacterial infections [51]. This pathway plays a central role in modulating the host’s immune response, thereby enhancing resistance to bacterial pathogens. Our study indicated that NL could dampen the insulin/IGF-1 signaling pathway, which in turn suggests the potential activation of DAF-16/FOXO. This activation is crucial for modulating immune responses, as evidenced by studies showing that DAF-16/FOXO plays a significant role in the immune regulation against bacterial pathogens. Specifically, the activation of DAF-16/FOXO has been linked to the induction of antimicrobial peptides and an enhanced resistance to infections in models such as *C. elegans* [52]. These insights underscore the importance of NL’s impact on the insulin/IGF-1 pathway not only in lifespan regulation but also in bolstering immune defenses and stress resistance.

The RNA-seq data indicate that NL treatment leads to the enrichment of differentially expressed genes across multiple metabolic pathways. The present study further verified that NL treatment could improve lipid degradation and diminish lipid synthesis metabolism, evidenced by the fact that lipid accumulation was reduced in NL-treated worms and the expression of genes pivotal to lipid catabolism was upregulated. These observations are consistent with the above findings that NL exerts its lifespan-extending effects probably by dampening both insulin/IGF-1 and TOR signaling pathways. As the main parts of the nutrient sensing system, insulin/IGF-1 and TOR signaling play important roles in lipid and carbohydrate metabolism. A general downmodulation of the insulin/IGF-1 and TOR pathway is associated with an improved mitochondrial oxidative metabolism and an increased energy expenditure, eventually leading to an enhanced catabolic activity of the adipose tissue.

Additionally, it has been documented that NL can activate the SIRT-1/AMPK/SREBP-1 pathway to regulate glucose metabolism [14]. AMPK (adenosine monophosphate-activated protein kinase) is a key enzyme in the regulation of cellular energy homeostasis. AMPK responds to low levels of ATP and, when activated, positively regulates signaling pathways such as fatty acid oxidation and autophagy to replenish cellular ATP while negatively regulating ATP-consuming biosynthetic processes, including gluconeogenesis, lipid, and protein synthesis. This is consistent with our data that NL treatment readjusts metabolic balance, activates catabolism, and inhibits anabolism. However, whether AMPK is involved in this process requires further experimental verification.

## 5. Conclusions

In conclusion, our study provides evidence that NL at 50 μM can extend lifespan by 15.9% and reduce lipofuscin accumulation in *C. elegans.* A total of 928 DEGs were identified in NL-treated worms by using a transcriptomic analysis. The analysis of DEGs indicated that NL’s longevity-promoting effects might be due to its modulation of gene expression in lipid metabolism and immune response, as well as its influence on key signaling pathways associated with aging. These findings provide valuable clues for future in-depth research on the mechanisms by which NL extends lifespan and may offer a scientific basis for the development of anti-aging interventions.

## Figures and Tables

**Table 1:** Longevity-associated DEGs in *C. elegans* treated with 50 μM NL.

Gene Name	Gene Description	F.C	Regulation
*mtl-1*	Metallothionein-1	5.94	Up-regulated
*lips-14*	-	2.30	Up-regulated
*ist-1*	Insulin receptor SubsTrate homolog	2.06	Down-regulated
*daf-2*	Insulin-like receptor	2.13	Down-regulated
*daf-15*	DAF-15	2.16	Down-regulated
*hsp-1*	Heat shock 70 kDa protein A	2.21	Down-regulated
*let-607*	-	2.27	Down-regulated
*tank-1*	Tankyrase-like protein	2.34	Down-regulated
*let-363*	Target of rapamycin homolog	2.80	Down-regulated

## Data Availability

The data presented in this study are available on request from the corresponding author.

## Supplementary Materials

The following supporting information can be downloaded at: https://www.mdpi.com/article/10.3390/metabo14120716/s1, Table S1: List of genes differentially regulated by NL treatment in *C. elegans*; Table S2: List of primers used for QPCR experiments; Table S3: KEGG pathways describing genes upregulated by NL treatment in *C. elegans*; Table S4: Gene ontology terms describing genes upregulated by NL treatment in *C. elegans*.
